# Nanomaterials Reinforced Polymer Filament for Fused Deposition Modeling: A State-of-the-Art Review

**DOI:** 10.3390/polym15142980

**Published:** 2023-07-08

**Authors:** Xinchun Luo, Hailong Cheng, Xin Wu

**Affiliations:** School of Chemical Engineering and Technology, Sun Yat-sen University, Zhuhai 519082, China; luoxch25@mail2.sysu.edu.cn (X.L.); chenghlong@mail2.sysu.edu.cn (H.C.)

**Keywords:** fused deposition modeling, polymer, nanocomposites, properties, application

## Abstract

For the past years, fused deposition modeling (FDM) technology has received increased attention in the applications of industrial manufacturing fields, particularly for rapid prototyping, small batch production and highly customized products, owing to the merits of low-cost, user-friendliness and high design freedom. To further expand the application potential and promote the performance of the as-manufactured products, many efforts have been spent on the development of suitable materials for FDM applications. In recent years, the involvement of nanomaterials in the FDM-based polymer matrix, which has been demonstrated with great opportunities to enhance the performance and versatility of FDM printed objects, has attracted more and more research interest and the trend is expected to be more pronounced in the next few years. This paper attempts to provide a timely review regarding the current research advances in the use of nanomaterials to reinforce polymer filaments for the FDM technique. Polymer composite filaments based on nanomaterials such as carbon nanotubes, nanoclay, carbon fibers, graphene, metal nanoparticles and oxides are discussed in detail regarding their properties and applications. We also summarized the current research challenges and outlooked the future research trends in this field. This paper aims at providing a useful reference and guidance for skilled researchers and also beginners in related fields. Hopefully, more research advances can be stimulated in the coming years.

## 1. Introduction

Additive manufacturing (AM), also known as 3D printing, is a promising technology which has the potential to reform the current industrial mode [1,2]. This technology was first developed by Stratasys in the late 1980s for the manufacturing of objects with complex geometries [3]. Basically, the realistic 3D objects are printed through the digital STL model files built from computer-aided design (CAD), based on which liquid or solid bondable materials are printed out with designed tracks and stacked layer by layer to build objects with desired structures [4,5]. Compared to traditional manufacturing techniques, AM technology has many advantages, such as a significant reduction of material waste, enhanced capability of manufacturing complex geometries, improved product performance and cost savings [6]. As a result, additive manufacturing technology has received a great deal of attention in the last decade or so and has been widely used in the fields of aerospace [7], automotive industry [8], medical [9,10], bioengineering [11], electronic sensing [12] and so on.

Currently, the main AM technologies developed include fused deposition modeling (FDM), selective laser sintering (SLS), stereolithography (SLA), inkjet printing (IJP) and layered solid manufacturing (LOM) [13]. According to the ISO/ASTM 52900:2015 standard, these AM technologies can be categorized into seven types depending on the forming principle and printing material, namely material extrusion (ME), photopolymerization forming (VP), directed energy deposition (DED), material jet (MJ), binder jet forming (BJ), powder bed fusion forming (PBF) and sheet lamination (SL) [14]. Each AM technology process has its own characteristics as well as application scenarios in terms of printing efficiency, accuracy, material type, printing speed and finished surface smoothness, and users can choose AM technology process according to their needs, greatly enriching the freedom of 3D printing technology.

**Figure 1:**
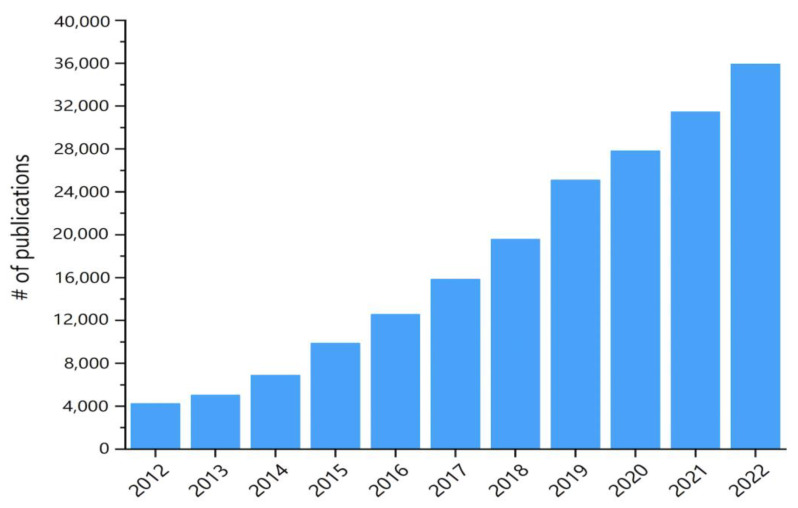
Total number of publications related to Additive Manufacturing/3D-printing since 2012 (data from Web of Science, 2023).

Since the advent of AM technology in the 1980s [3], a lot of research efforts have been spent on relevant fields to provide solutions for the existing issues in different application needs. Figure 1 shows data on the number of AM technology-related publications counted on the Web of Science since 2012, showing an exponential growth trend. AM technology has become an important manufacturing tool in modern manufacturing, particularly for rapid prototyping, small batch production and highly customized products. It was indicated that the future manufacturing mode is expected to be changed dramatically under the impetus of the AM technology revolution [15,16].

Fused Deposition Modeling, as one of the most widely used AM technologies, relies on thermoplastic polymer filaments as raw materials, which are fused in the heating chamber and deposited onto the substrate in a layer-by-layer manner to create the desired 3D objects. There are many engineering plastics available to be applied in the FDM technique. Due to its low cost, ease of use and high freedom of design capabilities, FDM technology has been widely used in areas such as prototyping, education, medical and personal consumer markets [17,18,19,20,21].

However, the current polymer materials available for FDM 3D printing generally have some limitations for their wide application potential, including insufficient mechanical properties, poor heat resistance, low electrical conductivity or high production cost, which would affect the print quality and the further expansion of the application scenario. For some application conditions, these limitations are fatal [22,23,24]. To address these limitations, many solutions have been proposed. In recent years, researchers have explored ways to improve the performance of polymeric materials by adding nanomaterials. Among different kinds of nanomaterials, commonly used nanofillers including carbon nanotubes (CNTs), graphene, metal nanoparticles (MNPs) and oxides, to name a few, have attracted significant interest due to their unique mechanical, electrical and thermal properties [25,26,27,28,29]. The incorporation of nanomaterials into thermoplastic polymer materials to prepare nanocomposites can not only effectively improve the mechanical properties, thermal conductivity and wear resistance of polymer materials, but also impart other special properties such as electrical conductivity and antibacterial properties [30,31]. In addition, nanomaterials may improve the processing capability of polymer materials, such as improving the extrusion stability and reducing the surface roughness of 3D printed composite filaments, thus improving the manufacturing precision and quality without significantly increasing the manufacturing costs. Furthermore, nanomaterial-reinforced polymer filaments can also be diversified in terms of material properties and functionality by adjusting the type and content of nanomaterials to meet the needs of different fields. With the development of advanced technology, new techniques and methods for the preparation of nanomaterials are emerging such as chemical vapour deposition, ion beam-assisted deposition, liquid phase stripping and electrospinning to name a few. The applications of these new technologies have made the preparation of nanomaterial-reinforced polymer filaments simpler, more efficient and more economical. As a result, the addition of nanomaterials into thermoplastic polymers for performance modification has become the focus of current research regarding FDM 3D printing technology.

This review will focus on recent research advances in the use of nanomaterials to reinforce polymer filaments including the preparation methods of different types of nanocomposites, their modifications and their applications in different fields. The properties and applications of nanocomposite filaments fabricated by FDM, especially polymer composite filaments prepared on the basis of nanomaterials, such as carbon nanotubes, nano clay, carbon fibres, graphene, metal nanoparticles and oxides, will be described in detail. We will also point out the current research challenges and outlook on the future trends in this field. The aim of this paper is to provide a useful reference and guidance for researchers in related fields.

## 2. Fused Deposition Modeling and the Filament Materials

### 2.1. Introduction of Fused Deposition Modeling

Material extrusion, also known as fused filament fabrication (FFF) or FDM process, is one of the most popular and widely used techniques in AM technology. This review focuses specifically on FDM technology, which is described in full below.

**Figure 2:**
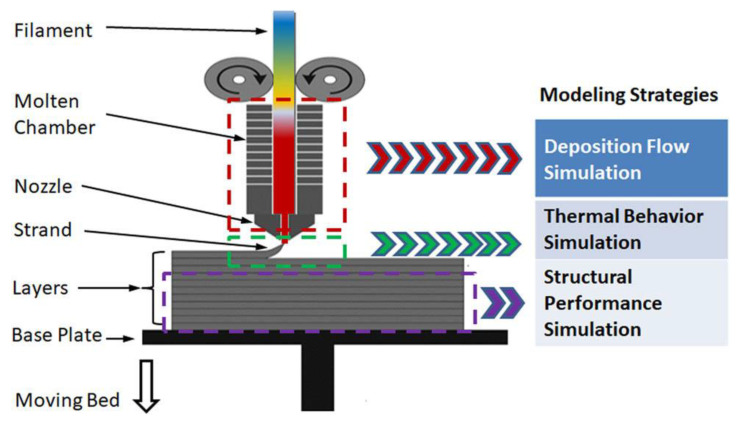
Schematic of the working principle for FDM process [39].

In 1988, Scott Crump invented the FDM technique and funded the company Stratasys [32,33]. This technique is similar to toothpaste squeezing and is simple and low cost to operate and maintain [34]. In the fused deposition modelling method, the first stage is to create a 3D geometry model of the desired part using a computer modelling software (CAD or Solidworks) and then convert the designed geometry model into a readable format by the FDM 3D printing machine, i.e., STL format. In the next step, the 3D geometry model in STL format is sliced into multiple layers using slicing software, followed by layer-by-layer fused deposition controlled by a programming language (G-Code code) to build the 3D models [35,36,37,38]. Specifically, the FDM process, shown in Figure 2, begins by loading a roll of filament into the printer and when the nozzle reaches the temperature as required, the filament is pressurized and extruded into the nozzle and melted in the nozzle. The extrusion head of the FDM printer is connected to the printer’s 3-axis system, and by walking a thin strip it first moves to a predetermined position in the *X* and *Y* directions of the print platform and then completes the printing of a layer. Thereafter, the table drops in thickness by one layer in predetermined increments along the *Z*-axis direction and the material is extruded and deposited on top of the previous layer. This layer-by-layer deposition process would be repeated and eventually realize the formation of the final products. In particular, the cooling rate of the material can be accelerated during the printing process by installing a cooling fan inside the 3D printer to achieve better printing results. Finally, the support is removed after printing and surface treatment is applied to obtain desired roughness.

The strength of a part constructed by the FDM process depends on the binding force between two successive layers. Sufficient thermal energy is required to activate the surface of the previously deposited layer and initiate adhesion between the activated surface and the newly deposited layer. The performance of the final constructed model (extrusion accuracy, model surface roughness, and mechanical properties) is strongly influenced by the FFF process parameters [40,41,42,43,44].

The main advantages of the FDM technique are as following:

(1) Low maintenance cost and safe system operation due to the simple construction principle and easy manipulation of the hot melt extrusion system.
(2) The forming speed is high, and the products produced by the fusion deposition method do not require the process of reworking scrapers as in SLA.
(3) No chemical changes in the raw material during the forming process, so that the warpage and deformation of the parts are small.
(4) High raw material utilization rate and rich material sources.

FDM printers are popular at home or in the office because of their simplicity, ease of use, good adaptability to many materials, miniaturization and ease of operation. Many companies have invented different types of 3D printers for desktop use based FDM principle. FDM printers allow individual users to customize various parts.

### 2.2. Preparation of Filament Materials

**Figure 3:**
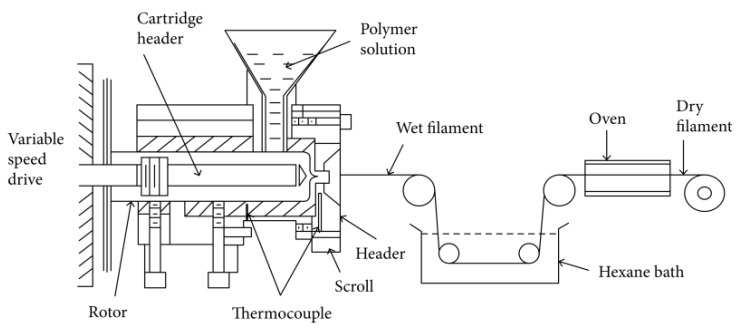
Schematic representation of the general filament extruder [45].

The FDM printing filaments are generally produced and manufactured by screw extruders, which include twin-screw type and single-screw type. The extruder system adjusts the extrusion speed, raw material melting temperature and output to suit the needs of production for different types of roll filaments. As shown in Figure 3, the working principle is to melt and mix the solid material through the rotating screws of the extruder and deliver it to the nozzle of the heater, eventually forming a continuous filament. Specifically, the solid material to be processed is first fed into the hopper of the extruder and the screws begin to rotate, bringing the material into the barrel of the extruder, in which place, the screw continues to rotate; meanwhile, the heating chamber heats the solid material to melt it. Once the material has passed some distance between the screws, melt mixing begins to take place, which causes the bonds between the material molecules to break and recombine, resulting in a homogeneous mixture. During this process, constant pressure is also applied to extrude the material to the outlet of the extruder, where a cooling unit, laser caliper and traction winder are applied to finish the industrial manufacture of the desired 3D printable wires.

**Figure 4:**
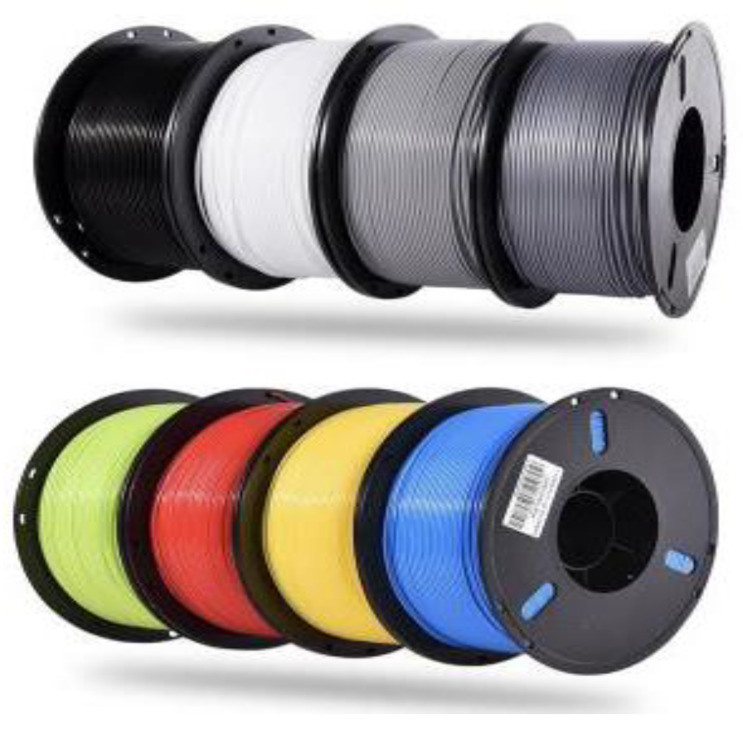
Typical FDM 3D printing filaments.

The typical filament rolls for FDM 3D printers produced by melt extrusion of solid granular materials are shown in Figure 4. At the same time, filament manufacturers add a variety of pigments to produce a wide range of colours for users to choose from, depending on the aesthetic needs of the public.

### 2.3. Materials for Fused Deposition Modeling 

For FDM technology, the materials used are thermoplastic polymer materials, which must be supplied in filament form and the requirements of good processability, a suitable modulus to viscosity ratio, low crystallinity and thermal expansion coefficient and excellent stability need to be met to ensure the compatibility with FDM technology. The range of polymer materials suitable for FDM processing is very limited compared to traditional polymer moulding techniques.

In the FDM technique, it is crucial that the users select the right polymer materials for the part to be manufactured. Different materials have diverse properties such as chemical resistance, flame retardancy, flexibility, corrosion resistance, minimal water absorption or optical transparency and careful consideration should be given by the users to choose the materials suiting their needs for different applications. To some extent, thermoplastic polymers are processable, adaptable and versatile enough to meet the requirements of a wide range of shapes and performance [46]. For instance, the mechanical properties reported in the scientific literature in terms of tensile strength range between 1.5 MPa and 150 MPa for thermoplastic polymers [47,48,49,50,51,52]. With the increasing popularity as well as the rapid development of FDM printers, polymeric materials with various applications are being created by researchers and commercialized by companies.

In this section, we outlined the commonly used polymer materials for FDM printing, as well as described the properties and current applications of these materials. Table 1 shows the typical thermoplastics used in the FDM process.

**Figure 5:**
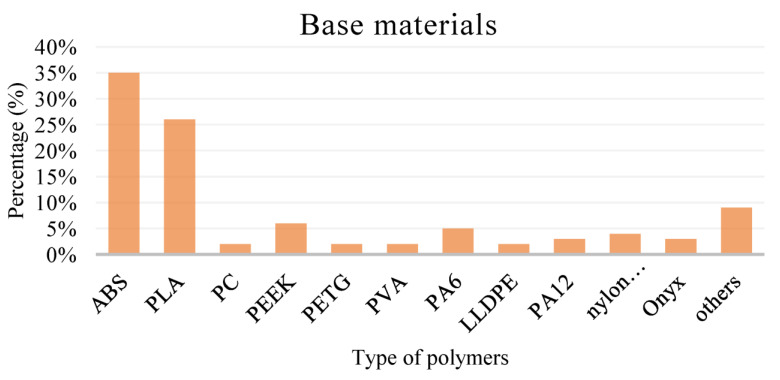
Current market shares for different types of polymer substrates for FDM technique [20].

The market shares for different types of polymer substrates used in FDM printing are shown in Figure 5. As can be seen, the most widely used FDM 3D printing material is ABS, with a 35% usage share, and the second most commonly used type is polylactic acid (PLA) at over 25%. In contrast, thermoplastic materials such as PC, PEEK, PETG, nylon and PVA are used relatively infrequently, at no more than 10%.

Acrylonitrile butadiene styrene (ABS) is the most widely used and earliest polymer printing filament material, with a moulding temperature of 180–250 °C, a printing temperature of 210–260 °C and a glass transition temperature of 105 °C. The base plate needs to be heated when printing [53,54]. ABS has many advantages, such as high strength, good toughness, impact resistance, good insulation properties, corrosion resistance, low temperature resistance, easy filamentation and colouring, etc. The quality of its printed products is stable, the strength is ideal and it is widely used in automotive, textile, electronic and electrical appliances and construction [55,56,57]. However, ABS also has some disadvantages. For instance, the obvious shrinkage characteristics during cold are easy to occur in the case of an uneven temperature field, as well as layer peeling and warping. In addition, a strong smell may be generated during the printing process [58].

Polylactic acid (PLA) is made from corn or sugar cane, which is fermented into lactic acid and eventually converted into polylactic acid [59]. PLA has good ductility, degradability, biocompatibility, great hardness of printed products, bright colors, transparent and glossy, fine appearance and no unpleasant odour during the printing process, making it one of the most popular raw materials for 3D printing [60,61,62]. The disadvantages of PLA are also obvious: poor toughness and impact strength, brittleness of printed products, low strength, poor dimensional stability and inability to resist temperature changes and deformation when the temperature exceeds 50 °C, which greatly limits the wide applications [63,64,65]. For this reason, researchers have spent lots of effort to improve the properties of PLA.

Polycarbonate (PC) is a thermoplastic resin with a carbonate group in its molecular chain. It is one of the most used thermoplastic engineering plastics because of its excellent properties [66,67,68]. PC has almost all the good characteristics of engineering plastics such as good impact resistance, odourless, high temperature resistance, bending resistance, high strength and also good flame-retardant properties. Especially, it has good printability and can be used in the FDM process to prepare high strength products [69]. However, PC also has some disadvantages: the colour is single and the colouring performance is not ideal; PC contains the carcinogenic substance bisphenol A, which precipitates at high temperatures and affects human health; the price is relatively high; the printing temperature is too high (over 300 °C), making it be unsuitable for most desktop 3D printers.

Poly(lactide) (PCL) is a biodegradable polyester with a low melting point (~60 °C), excellent biocompatibility and shape memory properties. As a result, PCL can be a good consumable for energy-efficient 3D printing, and its printed products are widely used in the medical field, especially for in vivo organ repair and cardiac stents. However, the mechanical strength of PCL is not good, even not as strong as PLA, therefore, the modification of the performance is highly desired.

Polyetheretherketone (PEEK) is a special engineering plastic with many significant advantages over other general engineering plastics, such as high-temperature resistance, excellent mechanical properties, good self-lubrication, chemical corrosion resistance, flame retardancy, peel resistance, irradiation resistance, stable insulation, hydrolysis resistance and easy processing; therefore, it has been widely used in aerospace, automotive manufacturing, electrical and electronics and medical and food processing [70,71,72]. At the same time, PEEK has similar elastic modulus, biocompatibility, chemical stability and radiation transmission to cortical bone, making it an excellent candidate for orthopaedic replacement. In addition, 3D printed PEEK products are rapidly developing as a representative of high-strength, high-temperature resistant and highly insulating polymers for high-end applications [73].

Polyethylene terephthalate-1,4-cyclohexanedimethanol (PETG) is a copolyester made from the copolymerisation of three monomers: dimethyl terephthalate (DMT), ethylene glycol (EG) and 1,4-cyclohexanedimethanol (CHDM) [74]. With a certain ratio of EG/CHDM, the introduction of cyclohexane units in the molecular chain reduces the regularity of the entire molecular chain, so it is a completely amorphous transparent copolyester with low crystallinity or even completely non-crystalline, excellent optical properties and good processability. In addition, it is also non-toxic and environmentally friendly, so it is recognized as a bio-based polyester material in line with environmental requirements [75,76,77]. When used as a 3D printing material, PETG has the advantages of both PLA and ABS, i.e., good mechanical performance, low printing temperatures, almost no odour, very low material shrinkage and good dimensional stability. PETG is widely used in the packaging of cosmetics, perfumes and other household products.

Other polymeric materials commonly used in FDM technology include polyamide (PA) or nylon [78,79,80], polyurethane (TPU) [81,82], polyetherimide(PEI) [83,84], Thermoplastic Elastomer (TPE) [85] and polypropylene (PP) [86,87]. These materials are widely used to print automotive parts, aircraft parts, toy models, medical items and many other daily-life products. However, the printed parts based on a single polymer filament strip often suffer from poor mechanical properties, lack of functionality and a tendency to warp. As a result, they are often blended with other materials to improve the processability, reduce costs and extend the application potential. A large number of studies have been carried out to modify the polymer matrix by adding micro and nano fillers (e.g., non-metallic and metallic materials) and to develop composite filaments for FDM processing to improve the anisotropy of FDM printed parts, as well as to improve the mechanical properties and dimensional accuracy and to give them certain functions, such as electrical and thermal conductivity and biomedical properties, which can endow FDM printed parts with high performance and abundant functionalization, expanding the application areas. Currently, most of the polymer fillers used for FDM printing are carbon-based fillers, including carbon nanotubes (CNT), nano-clay (NC), carbon fibres (CF), graphene, etc. In addition, metal nanomaterials and oxide nanoparticles filled with polymer materials for modification are also increasingly attracting the researchers’ attention. The following sections will detail the latest advances in composite materials applied to FDM from the aspects of carbon-based fillers, metal nanoparticle fillers and oxide nanoparticle fillers, respectively.

## 3. Polymer/Carbon Nanocomposites Based Modification and the Applications

### 3.1. Polymer/CNT Nanocomposites Based Composite Filaments

FDM printing technology has great advantages over traditional moulding techniques (injection moulding and compression moulding), as it can produce many parts more cheaply and flexibly, and the processability and properties of the nanocomposites made from the same raw material are significantly better than those of traditionally moulded materials, so FDM printing technology has great potential for thermoplastic nanocomposites [88,89]. In order to realize this advantage, the development of materials with excellent properties is a key factor. Compared to conventional conductive fillers such as carbon black [90], CNT fillers can achieve better performance in composites even with lower concentrations. Carbon nanotubes are tubes of layered carbon atoms hybridised in the form of sp^2^, which have unique mechanical, thermal and high electrical conductivity and are, therefore, ideal reinforcement for polymer-based materials. The development of CNT nanocomposites for wearable electronics applications is currently attracting significant research interest [91].

For FDM technology with newly designed composite filaments, there are many limiting factors, one of the major problems is the agglomeration of nanoparticles during the filling process, which leads to reduced functions of the added fillers and clogging of the nozzles. To solve the problem of nozzle clogging, Gnanasekaran et al. [92] used a commercial desktop printer to prepare unconventional polyester nanocomposites (CNTs, graphene-based polybutylene terephthalate). By optimizing the particle size value and distribution of the conductive filler, as well as parameters and conditions such as printing temperature, printing rate and substrate temperature, the nozzle clogging problem that usually occurs when printing nanocomposites by FDM technology was overcome. In addition, a comparison between polybutylene terephthalate/graphene and polybutylene terephthalate/CNTs prepared by FDM technology shows that FDM technology is more advantageous in the preparation of polybutylene terephthalate/CNTs, and the printed structures have great surface strength properties in addition to excellent electrical conductivity.

Similar work was conducted by Dul’s group [93], who explored the preparation of CNTs/ABS composites using ABS as a matrix. On the one hand, they optimized the machine control parameters during the printing process, and on the other hand, they used a completely solvent-free process for the preparation of the raw material, which was suitable for use with FDM technology. The results show that the tensile modulus, strength and thermal stability of the printed components have been significantly improved. In addition, the conductivity of the printed components was largely enhanced by the effective conductive network constructed by the FDM technology. More importantly, at a concentration of 8% (mass fraction) of carbon nanotubes, the FDM technology is still viable and no nozzle clogging occurs. However, the case of higher CNT concentrations is not described by Dul et al. By conducting similar experiments with FDM-printed ABS/CNTs composites, Dorigato et al. [94] demonstrated that when the CNT concentration reached 15%, the printed component was so brittle that it fails before it reaches the yield point, although there was a certain increase in the elastic modulus. In addition, the melt index tests indicated that the composite with 15% CNTs concentration was too viscous to be tested under the given conditions. Thus, the above two studies show that CNTs concentrations of 15% and above would make the FDM printer more prone to clogging and result in inferior product performance. Of course, in addition to controlling the concentration of filler particles to solve the nozzle clogging problem, the setting of printer parameters should not be neglected. Materials with a higher storage modulus need to be 3D printed at a slower rate or at a higher nozzle temperature. However, if the print temperature is too high or if the hot end is supplemented by a rather slow print rate, the residence time of the polymer would be too long and polymer degradation may occur, often destroying the printed structure.

Although nozzle clogging can be a problem when using composite materials for FDM technology, the advantages are obvious, especially in terms of the conductivity modification of the composite material.

**Figure 6:**
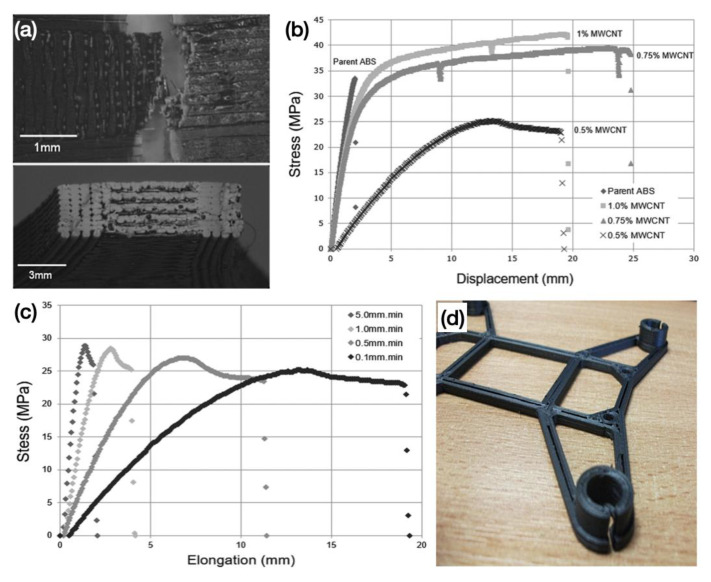
(**a**) SEM images of the fractured surface from the side and cross section of the as-printed 1% MWCNT nanocomposite. (**b**) Mechanical strength properties of the nanocomposites with 0.5, 0.75 and 1% MWCNT at a loading speed of 0.1 mm/min. (**c**) Mechanical strength properties of 0.5% MWCNT/ABS nanocomposite under different loading speeds (0.1 to 5.0 mm/min). (**d**) FDM 3D printed nanocomposite-based four-axis drone chassis [96].

In a separate study, Tsiakatouras et al. [95] conducted a comparative study of the mechanical properties of the samples prepared by conventional manufacturing methods, including injection moulding, and the FDM technique by adding a certain amount of CNTs to ABS resin as the consumable. The results showed that the FDM technique could improve the mechanical properties of the target, and more notably, the injection moulding method produced a more rigid structure, while the FDM method showed better manufacturing flexibility. Using the same consumables (ABS/CNTs), more research has been carried out to investigate the electrical properties of these materials. Thomas’ group [96] prepared 3D-printed components by dispersing multiwalled carbon nanotubes (MWCNTs) at different concentrations into an ABS polymer matrix. The results showed that the printability of the composites was not significantly reduced due to the high permeation threshold of the carbon nanotubes. The SEM images of the lateral and cross-sectional fracture interfaces produced after mechanical tensile testing of 1.0% MWCNT nanocomposites are shown in Figure 6a. The authors observed a degree of discontinuous fracture and ductile fracture surfaces in the test specimens, which were attributed to the increase in surface area caused by MWCNTs holding the polymer together under tension. In addition, the creation of an internal structure can be observed from the fractured surface of the printed specimen, where the longitudinal 3D printed shell structure will take on more strain energy owing to a continuous filament with no layer adhesion to hold the structure together. Tensile tests on MWCNT/ABS nanocomposites at 0.5%, 0.75% and 1.0% MWCNT concentrations showed that the nanocomposites at 0.75 and 1% concentrations presented better structural strength (Figure 6b). This indicates a good balance between the concentration of MWCNT and the polymer. Furthermore, tensile testing of the 0.5% MWCNT nanocomposites over a range of loading speeds exhibited a significant degree of work hardening (Figure 6c). In particular, a CNTs ratio of 0.75% resulted in a significant increase in the electrical conductivity of the printed components, with the most significant increase at 1%. Finally, the authors used the prepared nanocomposites to print a quad-axis UAV chassis (Figure 6d), demonstrating the unique advantages of FDM 3D printing. 

Dorigato et al. [94] were able to increase the electrical conductivity by nine orders of magnitude for the same consumables and the same CNTs concentration, and further by up to 13 orders of magnitude by increasing the filler concentration. In addition, the thermal diffusivity and thermal conductivity of the printed components were also significantly improved. In the study of electromagnetic shielding of non-magnetic composites, electrical conductivity is a very important indicator, and the level of electrical conductivity directly determines the electromagnetic shielding performance. Schmitz et al. [97] 3D-printed nanocomposite-based components with electromagnetic shielding properties by mixing carbon materials (carbon nanotubes and carbon black) as fillers into ABS. The results revealed that the electromagnetic shielding performance could reach 16 dB, which basically meets the standard of effective electromagnetic attenuation. A comparative study of the effect of different moulding orientations on the electromagnetic shielding performance was also carried out. The vertically concentric moulding direction can reduce the electrical conductivity of the component through micro-pores and cavities without substantially changing the mechanical properties of the printed component, thus achieving electromagnetic shielding.

Yu et al. [98] prepared carbon nanotube/PLA composite filament strips and performed FDM processing, and the results showed that the addition of carbon nanotubes had little effect on the mechanical properties of the printed products, mainly due to the large number of pore defects within the samples. However, the addition of CNT fillers greatly enhanced the electrical conductivity of the PLA-based composites, with a permeation threshold of 2 wt% (1.2 vol.%) observed for the PLA/CNT system.

**Figure 7:**
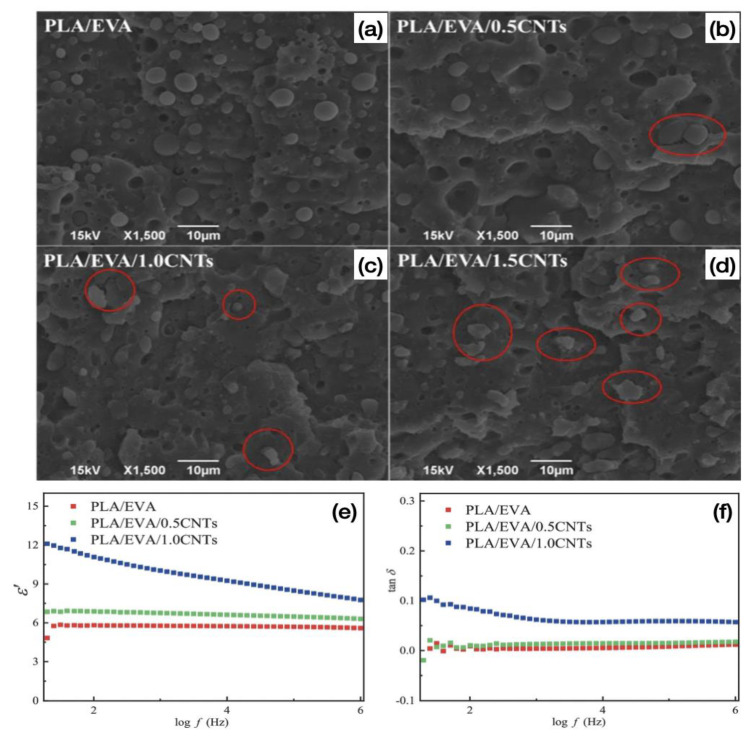
(**a**–**d**) SEM micrographs for the tensile fractured surfaces of the PLA/EVA composite. Frequency dependence of (**e**) ε’ and (**f**) tan δ for the PLA/EVA composites [100].

Spinelli et al. [99] investigated the effect of multi-walled carbon nanotubes (MWCNT) and graphene nanosheets (GNPs) on the dielectric properties of PLA composites. The results showed that the PLA composites with 12 wt% MWCNT greatly increased the dielectric field number from 3.7 before filling MWCNT to 5.35 × 10^3^ after filling, demonstrating that the addition of MWCNT would greatly improve the dielectric properties of PLA. On this basis, Wang et al. [100] prepared PLA/EVA-g-GMA/EVA/CNTs composites by the melt blending method and investigated the effect of carbon nanotubes on the interfacial morphology evolution and dielectric properties of PLA/EVA-g-GMA/EVA composites. The results showed that the filling of CNTs led to a slight decrease in the mechanical properties of the composites and that the introduction of CNTs as solid fillers led to a slow growth of PLA crystals and a decrease in crystallinity, but the thermal stability of the composites was improved, with the thermal decomposition temperature of PLA increasing from 350.5 °C to 359.1 °C for 1.5 wt% CNTs of the composites. Figure 7a–d shows the SEM images of the tensile fracture surfaces of the PLA/EVA composites. The PLA/EVA blend without CNTs shows a typical island structure (Figure 7a), with EVA as the dispersed phase in the blend as regular spheres of different sizes, closely adhering to the continuous phase PLA. As can be seen from the red circle marks in Figure 7b–d, the particle size of the EVA dispersed phase decreases significantly with the addition of CNT and gradually deforms from a regular spherical shape to an irregular shape, which may be attributed to the increase in viscosity of the composite due to the introduction of CNT. The composites exhibit fibrous tensile fracture surfaces with a typical ductile fracture regardless of the CNTs filling ratio, indicating that the addition of carbon nanotubes endows the PLA/EVA/CNTS composites with good ductility. Most importantly, only a small amount of CNTs was needed to significantly improve the dielectric properties of the composites. As shown in Figure 7e,f, the introduction of CNTs resulted in a significant increase in the dielectric constant (ε’) of the composites, especially for the composites with 1% CNTs, the dielectric constant (ε’) of the composites increased from 5.81 to 11.08, which is twice as high as that of the PLA/EVA blends. In addition, the lowest value of tan δ of 0.009 was obtained for the PLA/EVA blend at 100 Hz, and the tan δ of the PLA/EVA/1.0CNTs composite was raised to 0.084 with the addition of 1 wt% CNTs, which demonstrates that the addition of a small amount of CNTs resulted in a relatively high dielectric property of the composite. Combined with the SEM microscopic characterization of the tensile fracture surfaces in Figure 7a,d, it can be seen that the interfacial bond between PLA and EVA decreases with the addition of carbon nanotubes, leading to an increase in interfacial defects and leakage currents and ultimately an increase in tan δ values. The authors also investigated the tensile strength and elongation at break of PLA/EVA-g-GMA/EVA/CNTs composites, which reached 42.7 MPa and 70.5%, respectively.

Silvia et al. [101] prepared conductive composites of TPU/ CNTs by melt blending and showed that TPU/CNTs composites containing 1 wt% CNTs formed a conductive network and achieved the transformation from insulating to conducting materials. The properties of TPU/CNT composites such as conductivity, complex viscosity, energy storage modulus, loss modulus and EMI SE were enhanced with increasing content of CNTs conductive fillers. Kim et al. [102] successfully printed CNT/TPU nanocomposite filaments using FDM techniques based on the countermeasures of Sílvia et al. and fabricated 3D multi-axis force sensors.

**Figure 8:**
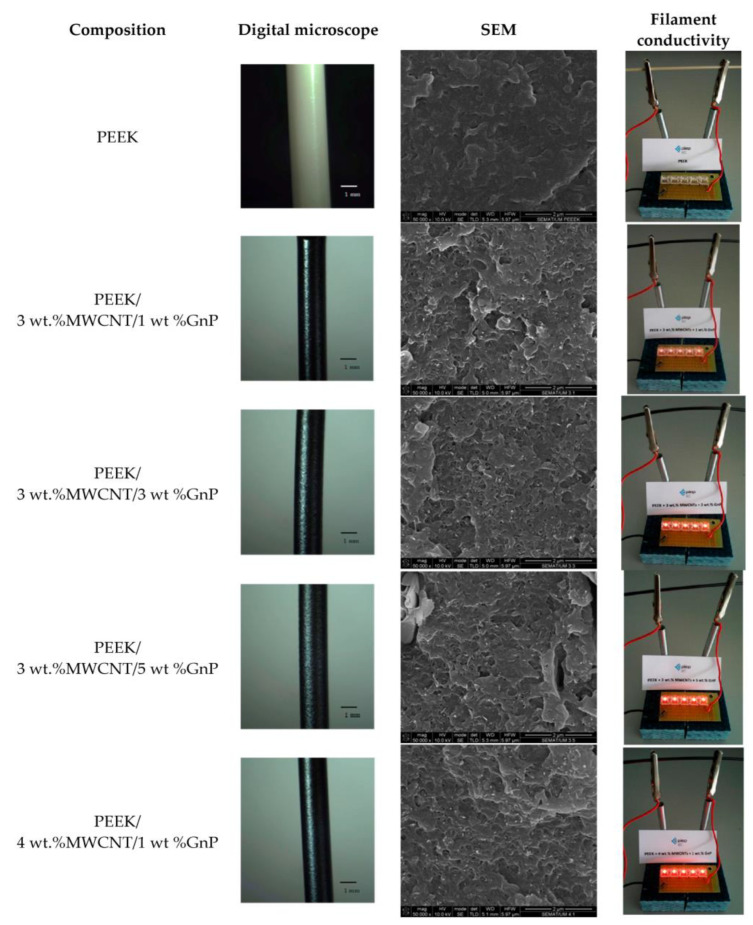
Digital microscope images, SEM micrographs, and illustrations of the electrical conductivity of the selected samples of PEEK and PEEK/CNT/ GnP extruded filaments [103].

Goncalves et al. [103] prepared PEEK nanocomposite filaments by adding CNTn and graphite nanoflakes to PEEK materials using fusion hybrid fabrication. The authors investigated the PEEK/CNT/GnP nanocomposite filaments with different CNT and GNP contents and Figure 8 shows the corresponding digital microscopy images, SEM of the cross-section and images of the intensity of the lit LED light for PEEK and PEEK/CNT/GnP filaments. The nanocomposite filaments were prepared by melt extrusion. The results show that for nanocomposites with 3 wt% CNT fillings, the addition of GnP up to 5 wt% does not affect the smoothness or diameter tolerance of the nanocomposite surface, despite a total carbon nanoparticle content of 8 wt%, which may be attributed to the lubricating effect of graphite. The filaments produced by a twin-screw melt extrusion were broken at low temperatures and SEM observation of the cross-section of the filaments showed a uniform distribution of filler in the composite as well as good wetting of the nanoparticles with PEEK. In particular, the increase in light intensity of the LED (withstanding 17 volts) with increasing MWCNT/GnP content provides evidence that all nanocomposite filaments with a CNT content of 1–5 wt% are within/above the electrical leakage threshold. Moreover, the DC bulk conductivity of the PEEK/MWCNT nanocomposites gradually increased as the concentration of MWCNT increased. However, the concentration of CNTs in the PEEK nanocomposites cannot reach as high as it could be; the actual maximum loading was 6 wt% with a DC volume conductivity of 12.22 S/m. When the concentration exceeds 6 wt%, abnormalities in the rheological behaviour of the composite would happen, preventing the FDM printing process. Finally, the authors prepared PEEK nanocomposites with good mechanical properties and high thermal conductivity in addition to the improved electrical conductivity.

### 3.2. Polymer/Nano Clay (NC) Based Composite Filaments

Nano clay is one of the most studied nanoparticles used in the production of composite materials. Nano clay is a natural mineral belonging to the smectite family, among which montmorillonite (MMT) is probably the most studied [104]. Nano clay composites have better properties than pure polymers, and the addition of nano clay can be a way to achieve enhanced mechanical strength, modulus and thermal deflection temperature [104]. Examples include silica, which have greater thermoplasticity and improved handling and performance qualities among thermoplastic polymers and polyhedral oligomeric siloxane (POSS)/PLA nanocomposites, which have increased flexural strength (by 22%), flexural modulus (by 9%) and toughness (by 17%) compared to pure PLA.

Coppola et al. [105] prepared PLA/layered silicate nano clay composites for FDM printers by melt mixing and screw extrusion. The results showed that the storage modulus of PLA composites filled with 4 wt% of layered silicate (Cloisite 30B) increased at 35 °C, for which PLA 4032D/C30B and PLA 2003D/C30B increased by 8% and 23%, respectively. In addition, the authors found that the addition of nano clay increased the thermal stability of the composites. The PLA/layered silicate nano clay composite samples printed using a FDM printer exhibited a higher modulus of elasticity than the neat PLA samples.

**Figure 9:**
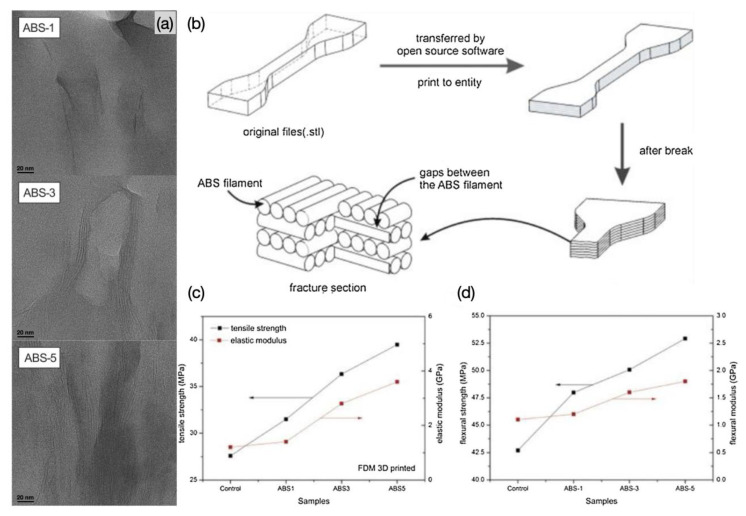
(**a**) Transmission electron microscopy images of the acquired ABS/OMMT nanocomposites, (**b**) illustrations of the fractured surfaces of printed parts, (**c**) tensile and (**d**) bending properties of the printed ABS/OMMT nanocomposites (The arrows are used to guide the corresponding y-axis of the data) [106].

Weng et al. [106] prepared ABS/OMMT nanocomposite filaments using a single-screw extruder and the samples printed using an FDM 3D printer was used for the evaluation and analysis of mechanical and thermal properties. The authors prepared composites by melt blending three different loading mass fractions of OMMT (1 wt%, 3 wt% and 5 wt%) with ABS, respectively. The formation of an intercalated structure of OMMT in the nanocomposites was confirmed by TEM data (Figure 9a). The authors compared the mechanical properties of ABS/OMMT nanocomposites under the FDM process with those under the injection moulding process. The data showed that the mechanical properties of the samples prepared under the injection moulding process were better than those under the FDM process, as a result of the high void content and low level of polymer chain entanglement in the FDM printed parts (Figure 9b). The difference between the FDM printing process and the injection moulding decreases when the OMMT loading is 5 wt%. As shown in Figure 9c,d, the mechanical properties of the ABS/OMMT nanocomposites improved significantly with increased OMMT loading, and for the nanocomposites with 5 wt% OMMT addition, the tensile strength of the FDM printed samples increased by 43%, while the injection moulded samples showed an increase of 28.9%. Furthermore, the addition of OMMT nano clay resulted in higher thermal stability and lower linear thermal expansion ratios for ABS.

Meng et al. [107] prepared ABS nanocomposite filaments with different types of nanofillers by melt extrusion and printed samples using an FDM printer to evaluate the properties. The authors compared the effects of montmorillonite (MMT), multi-walled carbon nanotubes, calcium carbonate (CaCO_3_) and silicon dioxide (SiO_2_) fillers on the mechanical and thermal properties of ABS samples. The results showed that when 1 wt% of inorganic nanomaterials were added to ABS, the printed samples all exhibited better tensile strength, flexural modulus and strength, improved thermal stability and reduced mechanical anisotropy than pure ABS. In particular, the addition of MMT to the ABS matrix increased the tensile strength by 7.6 MPa and the flexural strength by 9.4 MPa, and the addition of CaCO_3_ reduced the mechanical anisotropy of ABS from 42.1% to 23.9%. In addition, they compared the properties of ABS nanocomposite samples fabricated by the FDM process and the injection moulding process, and the results showed that the mechanical properties of ABS samples fabricated by the injection moulding process were generally better than those of all tested samples fabricated by the FDM printing process.

Gao et al. [108] investigated the thermal conductivity and tensile properties of hexagonal boron nitride nanosheets (BNNSs)/ TPU nanocomposites. They prepared thermoplastic TPU composites with 10 wt%, 20 wt% and 30 wt% hexagonal BNNSs content and the composites were printed by FDM for the tested samples. The results show that BNNSs/TPU composites achieved greatly improved thermal conductivity and tensile properties, with thermal conductivity reaching 1.80 W m^−1^ k^−1^, which is 650.0% higher than that of pure TPU. The authors compared the performance of BNNS/TPU composites with 30 wt% BNNS added to generate 30 wt% BN/TPU composites. The tensile strength and elongation at failure of the 30 wt% BNNS/TPU composites were increased by 106.2% (δ~29.9 MPa) and 116% (ω~899%). In addition, they demonstrated that nano-sized BNNSs filled into TPU can achieve a better performance.

### 3.3. Polymer/Carbon Fiber (CF) Based Composite Filaments

Carbon fibres are made from carbonised fibres that have been treated with an epoxy coating and pressed and woven with graphite. Carbon fibres are high-temperature resistant, friction resistant, thermally conductive and corrosion resistant, fibrous in shape, soft and can be processed into various fabrics. The low density of carbon fibres results in high specific strength and modulus. The main use of carbon fibres is as reinforcing materials in composite with resins, metals, ceramics and carbon to make advanced composite materials [109,110,111,112]. Carbon Nanofibers are fibrous carbon materials made of multi-layered graphite flakes with diameters ranging from 10 nm to 500 nm and lengths ranging from 0.5 um to 100 um, which are quasi one-dimensional carbon materials between carbon nanotubes and ordinary carbon fibres. It is a new generation of dual-use materials for military and civilian use and has been widely used in various fields such as aerospace, transportation, sports and leisure goods, medical and mining, machinery and textiles.

In order to study the effect of carbon nanofibres on the modification of ABS materials, Shofner et al. applied FDM technology to print carbon nanofibre-reinforced ABS plastic nanocomposites. Compared with pure ABS plastic, the tensile strength and modulus of 3D-printed nanocomposites increased by 39% and 60%, respectively when 10% nanofibres were added. The dynamic mechanical properties test also showed a 68% increase in the stiffness of the nanocomposites [113].

**Figure 10:**
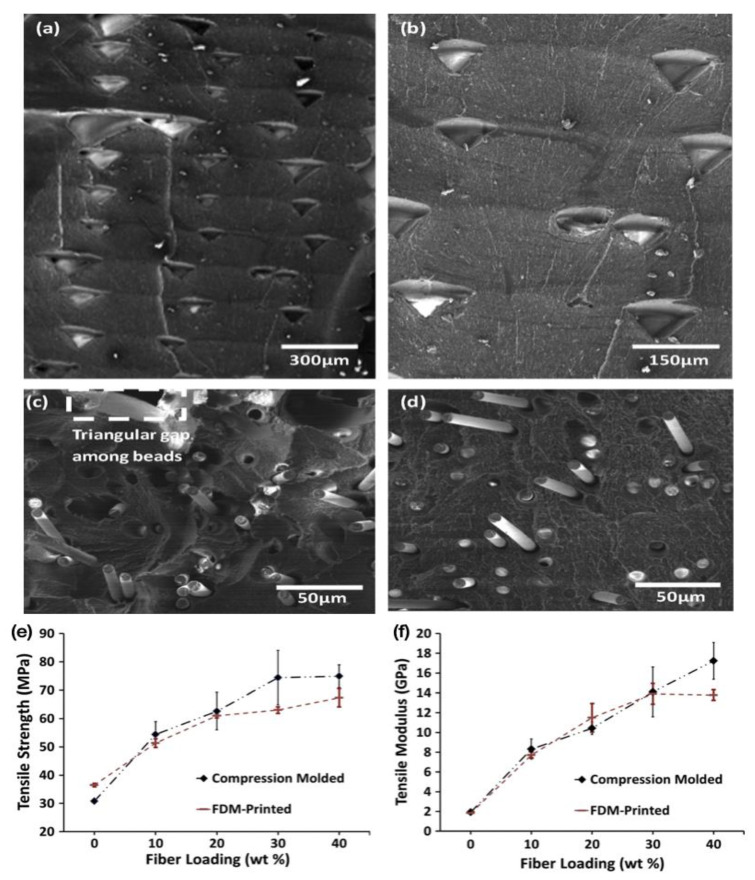
SEM micrographs of the fracture surface of (**a**,**b**) neat-ABS FDM-printed, (**c**) 10 wt% carbon fibre-loaded FDM-printed, and (**d**) 10 wt% CF-loaded compression-moulded ABS/CF composites. Protruding fibres are clearly separated from ABS, indicating poor fibre-matrix interfacial adhesion. Pore enlargement is evident around the fibres in the FDM sample, while no significant enlargement is seen in the CM sample. Effect of fibre content and preparation process on (**e**) tensile strength and (**f**) modulus, of ABS/CF composites [114].

Tekinalp et al. [114] examined the microstructure, processing properties and mechanical properties of ABS/CF reinforced composites manufactured by FDM and compression moulding techniques. ABS was reinforced with CF at 10, 20, 30 and 40 wt% and the filaments were extruded at 1.75 mm diameter. Specimens were printed at a layer thickness of 0.2 mm using a 0.5 mm diameter nozzle over a temperature range of 220 to 235 °C and at a bed temperature of 85 °C. The authors mentioned that ABS filaments containing 40 wt% CF could not be printed due to nozzle blockage during the FDM printing process. They found that the CM samples did not exhibit visible void content, while the FDM samples exhibited significant pore formation. SEM images of the fractured surfaces of the dog bone samples are shown in Figure 10a–d. In order to understand the mechanism of void formation, the FDM process was carefully investigated. Figure 10a,b shows the porosity of the printed pure ABS sample, where the pores consist of similarly oriented triangular voids. These voids are mainly gaps between the beads deposited during the printing process. It is important to note that as carbon fibres are added to the feedstock, voids within the beads begin to form (Figure 10c,d). As the voids within the beads create stress concentration points, they can cause the sample to fail at lower stresses. In addition, the increase in pore size around the fibres is evident in the FDM samples, whereas it is not evident in the CM samples. In particular, the authors compared the tensile strength and modulus of the specimens manufactured by FDM 3D printing and compression moulding (CM) processes, as shown in Figure 10e,f. The results show that in both processes the tensile strength increases with increased fibre content. It was observed that pure ABS samples prepared by the FDM process exhibited higher tensile strength than those prepared by CM, indicating that the FDM process could increase the molecular orientation of the polymer chains, and thus the tensile properties (Figure 10e). Figure 10f shows that the tensile modulus of the FDM- and CM-prepared samples based on nanocomposites filled with 0 to 30 wt% CF essentially overlap and increase almost linearly with increased fibre content. However, at a 40 wt% fibre loading, the FDM samples were difficult to fabricate due to the nozzle blockage that occurred with the FDM process, as a result only a thickness of a few layers can be printed. This thickness difference resulted in a difference in modulus between the FDM and CM specimens. The above results are attributed to the increase in the number of internal voids as the fibre content increases (Figure 10c), resulting in an early decrease in the enhanced strength of the FDM samples. Therefore, modifying/optimizing the blending process to minimize fibre fracture and modifying the FDM process to minimize the formation of internal voids may allow us to obtain stronger composite parts. Furthermore, as shown in the SEM micrographs of the fractured surfaces after tensile testing (Figure 10c,d), the fibres in both the FDM and CM samples were pulled out of the matrix, showing weak fibre–polymer interfacial adhesion, which also negatively affects the composite strength, and therefore, an improved interfacial adhesion could also significantly influence the mechanical properties of the FDM-printed parts.

In addition, Li et al. [115] analysed the flexural properties of PEEK/CF fibre-reinforced composites. The specimens were printed in both horizontal and vertical directions using ozzle and bed temperatures of 400 °C and 160 °C, respectively, and a raster angle of 45°/−45° with a layer thickness of 0.1 mm, as well as a printing speed of 15 mm/s and an air gap of 0.18 mm. The flexural properties of the vertically printed specimens were higher than those of the horizontally printed specimens. Both porosity and homogeneous nucleation were improved for PEEK with CF added compared to pure PEEK.

Spoerk et al. [116] investigated the anisotropy of the short carbon fibre (SCF) filled polypropylene (PP). SCF was mixed into PP with a content of 10, 15 and 20 wt%. Meanwhile, stabilizers and compatibilizers were also added to the compositions. The samples were printed with 0.25 mm layer thickness at 230 °C and at different orientation angles using 1.75 mm diameter filaments. This study concluded that the composites with 10 wt% SCF had much better properties compared to that of the 15 and 20 wt% SCF.

### 3.4. Polymer/Graphene Based Composite Filaments 

Graphene nanosheets (GNPs) are tiny stacks of graphene that can replace carbon fibers, carbon nanotubes, nanoclays or other substances. GNPs have excellent stretchability, strength and stiffness, as well as desirable functional properties such as high thermal and electrical conductivity. They are also light and easy to handle. Due to their unique two-dimensional structure, GNPs have a larger specific surface area, which makes them more effective in load-bearing reinforcement applications. For this reason, GNPs are considered to be an effective reinforcing agent in composites [117].

However, the relatively high specific surface area of nanoparticles leads to their more difficult dispersion in polymeric matrices. In addition, the incorporation of nanoparticles can substantially increase the viscosity of the material and make extrusion difficult. Zhang et al. [118] used chemical, low-temperature plasma and in situ methods to surface modify GNPs to investigate the effects of these modalities on the rheological properties of TPU/GNPs nanocomposites, as well as the mechanical properties, dimensional accuracy and surface roughness of FDM printed test samples. They prepared GNPs/TPU nanocomposite filaments by filling 3 wt% of surface-modified GNPs into TPU and printed test specimens with an FDM 3D printer. The results show that the modification of GNPs with an amphoteric surfactant (ADA) provides the best multifunctional performance for TPU. The pro-organic absorption between the fatty chains of the ADA and the TPU matrix leads to a homogeneous dispersion of the particles in the TPU matrix, providing a solution to the problem of agglomeration of graphene nanomaterials filled with polymers. The TPU/GNPs composites prepared by the chemical modification method using ADA exhibited higher electrical conductivity compared to the other two modification methods. Compared to the unmodified TPU/GNPs composite, the ADA-modified composite showed a 196% increase in electrical conductivity, and the FDM-printed test parts showed significant improvements in dimensional accuracy and mechanical properties, with tensile strengths of up to 69.79 MPa and elongation of 645%.

Dul et al. [119] prepared graphene/ABS composites with a graphene content of 4% by the solution method and melt-processed them to obtain composite filament strips suitable for FDM processing. In addition, the incorporation of graphene reduced the linear thermal expansion and creep of the material, improving the dimensional stability of the part.

**Figure 11:**
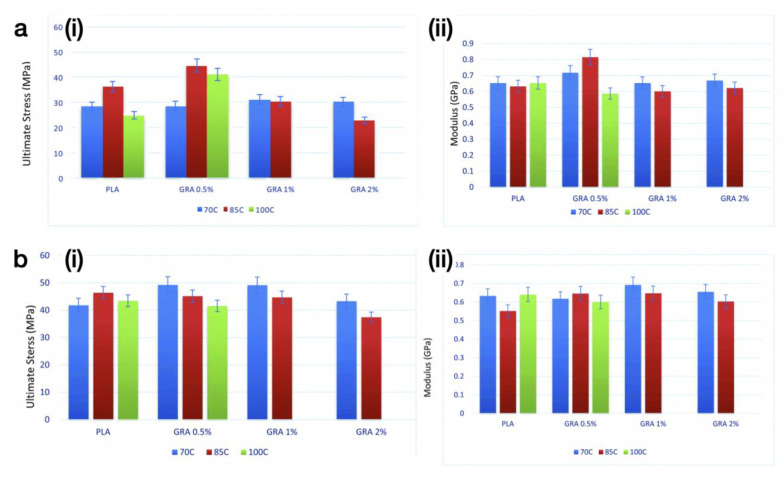
Ultimate stress of each sample printed in the (**ai**) *Z* direction, and (**bi**) *XY* direction. Modulus of each sample printed in the (**aii**) *Z* direction and (**bii**) *XY* direction [120].

FDM can effectively enable the printing of complex three-dimensional structures. However, it lacks the isotropic and robust mechanical properties required to perform large-scale manufacturing. Sahar et al. [120] provided a method to optimize the intrafilamentary interface and mechanical properties of FDM printed structures by filling PLA with graphene, which has high thermal conductivity, to develop multifunctional composites with improved FDM processability by enhancing the thermal conductivity of the thermoplastic material and investigated the effect of the composites on the thermal evolution, interfilamentous voids and mechanical properties of the fabricated samples during FDM 3D printing. Figure 11a show the ultimate stresses and moduli of PLA and PLA/graphene composites at different print bed temperatures in the *Z*-direction (Figure 11a) and *XY* direction (Figure 11b) for different graphene filling concentrations. The results show that at a lower graphene filling concentration (0.5%), the graphene/PLA composites exhibit better performance in both *XY* and *Z* directions. It showed that at lower graphene content (0.5%), the bonding between PLA printed filaments was improved, the interfilamentous gap was smaller and the FDM printed samples behaved isotropically. However, if the amount of filled graphene is too high, it can lead to slowly interfilamentous diffusion in PLA/graphene composites.

Prashantha et al. [121] investigated the mechanical and electromagnetic shielding properties of graphene/PLA printed parts and indicated that uniformly dispersed graphene significantly improved the dimensional accuracy and mechanical properties, which can impart good electromagnetic shielding properties to the printed objects.

Lu et al. [122] prepared PVA/GNP composites using ultrasonication and produced uniformly dispersed PVA/GNP nanocomposite filaments by screw extrusion. The FDM 3D printed parts were demonstrated with enhanced electromagnetic interference (EMI) shielding capability. The results showed that the incorporation of GNPs promoted the enhancement of hydrophobicity in the nanocomposites. Mechanical properties such as Young’s modulus, elongation at break and ultimate tensile stress of the PVA/GNP printed parts were optimized. In particular, the mechanical properties of the PVA/GNP nanocomposite printed parts filled with 8 wt% GNP were optimal with Young’s modulus of 49.1 MPa, ultimate tensile stress of 10.6 MPa and elongation at break of 128.4%. In addition, in the frequency range of 8–12.4 GHz, the PVA/GNP (8 wt%) printed part achieved its EMI shielding efficiency of 26–32 dB at a thickness of 2.43 mm. Compared with the pure PVA sample, the EMI performance of the nanocomposite was significantly improved to meet the needs of practical use.

Dingchun et al. [123] successfully prepared polyamide 12 (PA12)/GNPs nanocomposite filaments with different GNPs contents (2, 4, 6 wt%) using melt lamination and single-screw extrusion for FDM 3D printing. It was found that the nanocomposite filled with 6 wt% GNPs had a slightly reduced crystallinity and a suitable MFI value, giving the best performance compared to the cases of 2 wt% and 4 wt% GNP content. The addition of GNPs resulted in the PA12/GNPs nanocomposite a slightly reduced thermal stability, while a significantly improved thermal conductivity and elastic modulus. In addition, the thermal conductivity (K) and modulus of elasticity (E) of the test samples prepared by FDM 3D printing increased by 51.4% and 7%, respectively, compared to those prepared by injection moulding, due to the preferential alignment of GNPs along the print direction during the printing process. Furthermore, the ultimate tensile strength of the PA12/GNPS parts printed by FDM 3D printing was well maintained.

**Figure 12:**
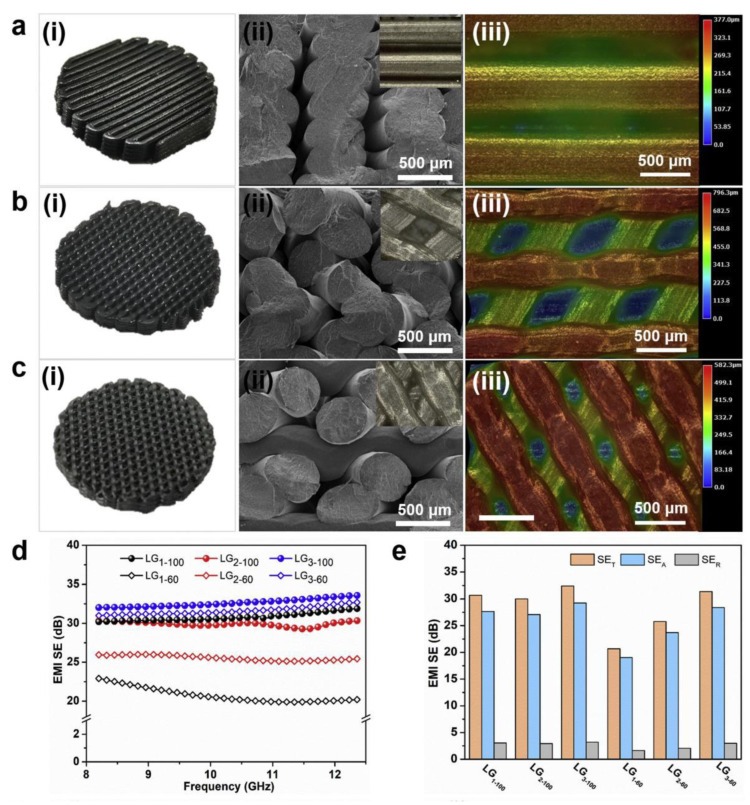
Effect of the printed porous structure on the EMI SE properties of LLDPE/GNPs parts: digital photos of (**ai**) LG_1–60_, (**bi**) LG_2–60_ and (**ci**) LG_3–60_ FDM printed parts, (**aii**, **bii** and **cii**) SEM images of the corresponding fractured surfaces, (**aiii**, **biii** and **ciii**) super-depth-of-field images of filament alignments. (**d**) EMI SE of the LLDPE/GNPs parts with different filling patterns (1–3) and different infill densities (60% and 100%). (**e**) Dependence of EMI SE_T_, SE_A_ and SE_R_ on the filling pattern and infill density at the frequency of 10 GHz [124].

Jingjing et al. [124] prepared LLDPE/GNPs composites and printed lightweight components with porous and complex geometries using an FDM printer. It was shown that the FDM-printed composite lightweight parts achieved outstanding EMI shielding of approximately 32.4 dB in the 8.2–12.4 GHz range. Furthermore, the porous lightweight parts they prepared exhibited superior EMI shielding at lower densities compared to other lightweight shielding parts in the literature. More importantly, they improved the mechanical properties of the composites using MW radiation technology, resulting in increased interfacial bonding strength between the LLDPE/GNPs composite filaments. Furthermore, in order to assess the effect of the FDM printed filling patterns on the EMI SE performance of LLDPE/GNPs printed parts, the authors designed three different 3D printed filling patterns (Figure 12ai,bi,ci). The SEM cross-section images (Figure 12aii,bii,cii) and super-field images of the surface (Figure 12aiii,biii,ciii) indicated that the deposited filaments with a porous structure (LG_3–60_) bind relatively more tightly, producing excellent interfacial strength. Figure 12d shows the EMI SE of the LLDPE/GNPs printed parts. The results show that the EMI SE for all test samples is in the range of approximately 30–32 dB at 100% fill density with very little variation, with the best EMI SE for the LG_3–100_. However, when the fill density is 60%, the different filling patterns show a significant difference regarding EMI SE. To clarify the source of the difference, the authors further investigated the SE_T_, SE_A_ and SE_R_ of LLDPE/GNPs printed parts with different filling patterns at 10 GHz (Figure 12e) and concluded that the difference in EMI SE was mainly induced by SE_A_, i.e., absorption. Their proposed preparation method is expected to be widely used in aerospace applications, smart devices and so on.

## 4. Polymer/Metal Nanoparticles (MNPs) Based Composite Filaments

Adding metal nanoparticles to thermoplastic materials to prepare multifunctional composite filaments is one of the current research hotspots in the field of 3D printing. In recent years, many researchers have conducted in-depth studies in this field and made some important progress [125]. Such filaments are made from a mixture of polymers and metal nanoparticles with diameters usually between 1 and 100 nm. Common metal nanoparticles include silver, gold, copper, nickel, iron, etc. They have unique physical, chemical and optical properties such as Surface Plasmon Resonance (SPR) effect, high surface area, high surface activity, etc. The addition of metal nanoparticles to polymer matrices can improve the mechanical, electrical and thermal properties of composites such as enhanced thermal conductivity, increased mechanical strength and improved electrical conductivity. In the field of 3D printing, metal nanoparticle composites are widely used to improve the properties and expand the applications [126].

In many industrial applications, interlayer fractures occur in FDM printed parts due to poor interfacial adhesion and low surface quality of the thermoplastic material [127]. Therefore, nanocomposites for FDM 3D printing were prepared by adding nanometallic fillers to enhance the industrial applications of these nanocomposites. Chen et al. [127] blended aluminium nanomaterials with PLA materials to produce air-cooled heaters with high thermal conductivity at low cost. These PLA/Al composites were FDM 3D printed and then surface treated with laser polishing, resulting in the printed parts with reduced surface roughness, increased storage rate, reduced loss angle tangency, increased tensile strength and modulus. On this basis, Chen also involved Cu nanomaterials into a PLA matrix to prepare Cu/ PLA nanocomposites. To improve the surface structure of the printed parts, laser polishing was used to melt the surface of the polymer substrate, resulting in a smoother surface [128]. The laser polishing treatment reduced the surface roughness of the FDM printed parts by more than 90% to 0.87 μm Sα using a 5 W laser at 200 um (ideal parameter). The PLA/Cu nanocomposites were demonstrated with strong interfacial adhesion and also significantly improved glass transition, as well as enhanced energy storage modulus, loss modulus, Young’s modulus (by 34.2%) and tensile strength (by 52.98%) [128].

In addition, silver nanometal fillers were able to improve the mechanical and antimicrobial properties of the composites. Bayraktar et al. [129] investigated the properties of nanocomposites by solution mixing silver nanometals into PLA. During composite preparation, the suspended silver nanomaterials were homogeneously aligned in the shear direction. TGA showed that silver nanofillers affected the degradation of the PLA matrix: they increased the degradation temperature and crystallinity before printing, while decreased Tg and the crystallinity after 3D printing without changing Tm. Overall, the silver nanometallic fillers increased the barrier to the degradation of PLA/Ag nanocomposites and also increased the antimicrobial properties, with both S. aureus and E. coli being 100% killed for the PLA/Ag nanocomposites with different concentrations of silver nanometallic fillers added [129]. Based on this, Podstawczyk et al. [130] prepared PLA/Ag nanocomposites by adding 0.01–5 wt% silver nanoparticles. The results showed that these PLA/Ag nanocomposites with different Ag concentrations showed excellent antibacterial properties against Escherichia coli, Pseudomonas aeruginosa and Staphylococcus aureus.

**Figure 13:**
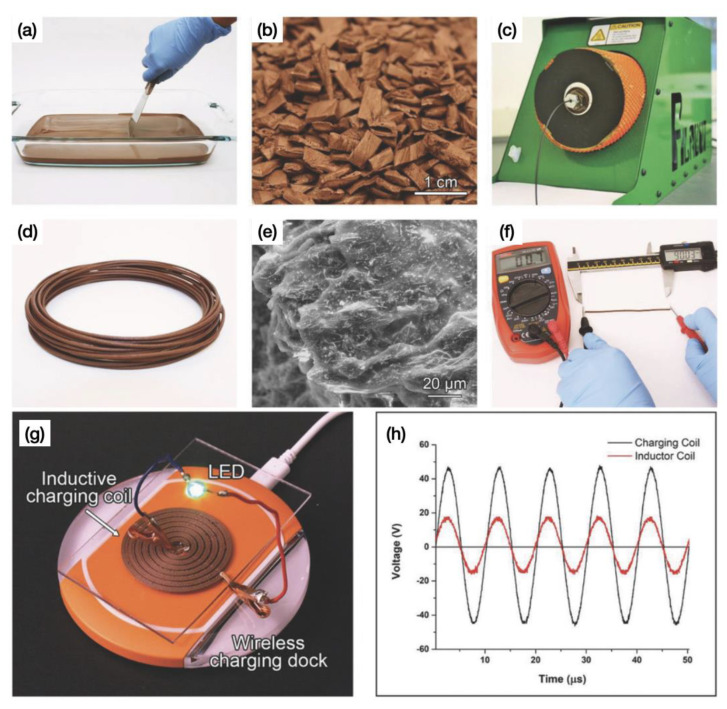
(**a**) Filament production started with a mixture of Cu-Ag NWs and PCL dissolved in DCM. (**b**) Drying of the solution-based solidified composite which was cut into uniform pellets. (**c**) Extrusion through a Filabot to form the filament. (**d**) The Filabot produced a coil of conductive filament similar in dimensions to other commercially available filaments used for 3D printing (diameter = 1.75 mm). (**e**) SEM image shows the dispersion of Cu-Ag NWs in the PCL filament. (**f**) A 90 mm length of filament with a diameter of 1.8 mm had a measured resistance of 0.7 Ω. (**g**) Demonstration showing how the Cu-Ag NW filament can be used to 3D print an inductive charging coil for wirelessly powering an LED. (**h**) Oscilloscope measurement of the transmitted (charging coil) and received (inductor coil) waveforms [131].

Similar to carbon-based fillers, metal fillers can also exhibit different shapes, usually nanowires, flakes or particles. Cruz et al. [131] produced composite conductive wires by adding 12 vol% Cu-Ag NWs (0.10 Ag:Cu mol ratio) to PCL dissolved in dichloromethane (DCM) (Figure 13a). The DCM was completely evaporated to give a solid composite. The composite was then cut into pellets (Figure 13b) and extruded into filaments using an extruder (Figure 13c,d). The SEM image shown in Figure 13e shows that the Cu-Ag NWs are uniformly distributed throughout the filament at this volume fraction and length scale. Moreover, the resistivity of the composite conductive PCL filament was 0.002 Ω·cm before FDM 3D printing (Figure 13f). This is an improvement of more than two orders of magnitude over various previously reported carbon-based fillers. And the 3D printed composite achieved good stability at 110 °C with excellent current densities between 2.5 and 4.5 × 10^5^ A m^−2^. In addition, they applied FDM technique to print a conductive coil for wirelessly powering of LEDs based on this Cu-Ag NW composite filament (Figure 13g). Figure 13h shows the measurement data of the transmit (charging coil) and receive (inductive coil) using an oscilloscope yielded voltage signal waveforms. A total of 40% of the input voltage was transmitted to the printed coil, which is consistent with the expected results for inductive differences.

Another highly conductive FDM filament was prepared by Tan and Low, who added nickel particles and Sn95Ag4Cu1 to the thermoplastic composite filaments of nylon-6 or high-density polyethylene (HDPE) by single-screw extrusion [132]. Conductivity values of 2.3 × 10^4^ Sm^−1^ or 31 × 10^4^ Sm^−1^ were achieved in HDPE and nylon-6, respectively. The authors studied a high metal loading of 30%-35% and they concluded that the melt viscosity was reduced due to the addition of the tin alloy compared to pure nickel particles.

In addition to the pure metal-based fillers, several groups have also examined metal/carbon fillers in different shapes. Wajahat et al. [133] decorated graphene sheets with magnetite nanoparticles and added them to hydroxypropyl cellulose to prepare conductive nanocomposite inks for extrusion-based 3D printing. With this conductive ink, objects with a conductivity of ≈580 S m^−1^ were produced. Due to the additional magnetic properties of this material, the authors suggested its use for 3D printing of magnet-guided cars, magnetic switches or EMI shielding. Xiang et al. [134] combined silver nanoparticles to prepare FDM printed strain sensors with a high sensitivity of 43260 at 250% strain and resistivity of ≈10^4^–10^6^ Ωm in the relaxed state, while Wei et al. [135] modified the PLA matrix using solvent-cast Ag-coated carbon nanofibres and 3D printed to prepare smart grippers with conductivity ≈210 kS m^−1^ from this material.

## 5. Polymer/Oxides Based Composite Filaments

Polymer/oxides-based composite filaments are a special type of FDM printing material, which is a composite material consisting of a mixture of polymer materials and oxide particles. This composite material can improve the mechanical properties, electrical conductivity and thermal conductivity of the printed objects, thus extending the scope of applications. Polymer/oxides-based composite filaments can be prepared in a variety of ways, including melt blending, impregnation and solution co-mingling. Different preparation methods affect the particle size distribution, dispersion and other properties of the composites. In addition to the preparation method, the type and content of oxide particles will also affect the properties of the composite. Common oxide particles include graphene oxide, copper oxide, aluminium oxide, zinc oxide, etc. The addition of these particles can improve the hardness, stiffness, wear resistance and other properties of the composite.

Graphene composites have excellent properties. However, it is prone to agglomeration, leading to a decrease in composite performance. To avoid graphene nanosheet aggregation, some researchers used graphene oxide (GO) with oxygen-containing functional groups on its surface. Wei et al. [136] were among the first to develop ABS composites for fusion deposition using graphene oxide. The results showed that the oxygen-containing functional groups resulted in GO better dispersion performance than pure graphene. ABS and graphene oxide were more adequately mixed, as shown by electrical conductivity tests on the composites, that is when the graphene oxide filler increased from 0.4 wt% to 5.66 wt%, the electrical conductivity of the ABS/GO nanocomposites increased from 1.78 × 10^−7^ S/m to 1.05 × 10^3^ S/m.

Yamamoto et al. [137] investigated the mechanical properties of graphene oxide/ABS nanocomposites at very low concentrations of graphene oxide (0.02, 0.04 and 0.06 wt%). In order to achieve better GO dispersion, the composites were subjected to sufficient solution mixing and ultrasonic treatment prior to FDM 3D printing. The results showed that the nanocomposites had improved strength and stiffness. Although all printed samples exhibited brittle failure, the composites gained improved ductility and toughness as the GO filler content increased. In particular, the GO/ABS nanocomposite samples with 0.06 wt% GO showed a 29% increase in elongation at fracture and a 55% increase in toughness when printed in the P-direction.

**Figure 14:**
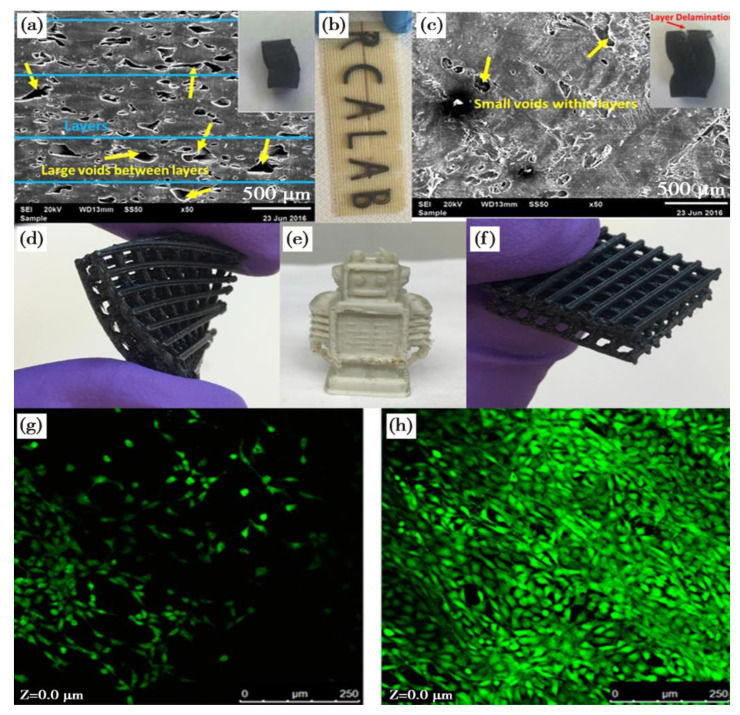
FDM printed biocompatible polyurethane/poly (lactic acid)/graphene oxide nanocomposites: (**a**,**c**) Microstructural morphology of the printed sample. (**b**,**e**) Macro morphology of the printed products. (**d**,**f**) As-printed micro-lattice. (**g**,**h**) Cell culture on the printed sample (green colour indicates live cells) [138].

Chen et al. [138] homogeneously mixed a mixture of graphene oxide with thermoplastic polyurethane and polylactic acid in a solvent, and then used the FDM technique to print the graphene oxide nanocomposites, which outperformed the nanocomposites without graphene oxide in mechanical and thermal stability performance. The mechanical properties of this material were oriented in the printing direction. The material was found to be compatible with active cells in biological cell tests, as shown in Figure 14, demonstrating that the application of 3D printed nanocomposites in biological tissue engineering, especially in biological scaffolds, will be a reality and become a potentially disruptive technique in the near future.

Jedsada et al. [139] prepared ZnO/CNT/PLA nanocomposites by filling ZnO nanorods into CNT/PLA composites and successfully printed electrodes with an FDM 3D printer for use as cyclic voltammetric sensors in electron tongue analysis. The results show that the ZnO nanorod filler can modify the electrical and electrochemical properties of the CNT/PLA nanocomposites, significantly altering the thermoelectric properties of CNT/PLA and moderately affecting the electrical conduction of CNT in PLA at room temperature. The FDM 3D-printed electrodes based on CNT/PLA/ZnO nanocomposites are electrochemically stable and can perform multiple CV measurements. PCA analysis can effectively distinguish CV signals from various substances, including different concentrations of K_4_Fe (CN)_6_, H_2_O_2_ and NADH, thus demonstrating the possibility of applying FDM 3D-printed CNT/PLA-based electrodes in electronic tongue sensors.

Anton et al. [140] added alumina (Al_2_O_3_) nanoparticles to PLA to make Al_2_O_3_/PLA composite filaments and used FDM to print test samples for tensile testing as well as to print ‘scraper’ type products. Based on particle size distribution analysis and fracture probability analysis, they showed that the addition of 10^−5^ to 10^−6^ mm sized Al_2_O_3_ particles effectively reduced the fracture probability of the test samples. However, Al_2_O_3_ particles in the range of 10^−1^ to 10^−2^ mm cannot change the fracture probability, maintaining a complex distribution probability curve and increasing the minimum value.

In order to prepare wearable thermoelectric devices for the conversion of bulk thermal energy into electrical energy, Afifeh et al. [141] added CuO nanoparticles to multi-walled carbon nanotube/polypropylene composites to prepare CNT/CuO/PP nanocomposites. FDM-printed filaments were synthesized by the nanocomposites and then printed on fabrics using the FDM printer. They investigated the electrical conductivity and Seebeck coefficient of the masterbatch, CNT/CuO/PP nanocomposite printed filament, and fabric-printed layer, respectively. The results showed that the electrical conductivity of the masterbatch, nanocomposite filament and fabric printed layers were 1.667 × 5^−10^ s/cm, 2.586 × 6^−10^ s/cm and 3.42 × 7^−10^ s/cm, respectively. In addition, the Seebeck coefficients of the polymer layers printed on masterbatches, nanocomposite filaments and fabrics were 489 μv/k, 430 μv/k and 220 μv/k, respectively.

Polymer/oxides-based composite filaments are currently being used in a wide range of FDM printing applications. They can be used to manufacture high-strength structural parts, electrically conductive structural parts, thermally conductive structural parts, etc. In addition, these composites can be used to manufacture 3D-printed products with special functions, such as sensors, capacitors, etc.

## 6. Conclusions and Future Work

Additive manufacturing is one of the most important driving forces for the fourth industrial revolution. The usage of AM has been rapidly developed in many industries. As one of the most popular AM techniques, FDM gives designers endless space to imagine. FDM allows a variety of complex designs to be produced into physical objects at a much lower cost than traditional manufacturing. This paper attempts to provide a comprehensive summary of the common polymers used for FDM and the latest advances in the development of polymeric nanocomposites. The development of polymer nanocomposites for FDM offers a way to extend the portfolio of material types, making it possible to produce versatile parts with more design flexibility. The role of FDM should not be to completely replace traditional manufacturing processes but to complement and make them more robust. To take full advantage of FDM, new approaches for nanocomposite design should be developed, based on which a wide range of applications for FDM processes in industries such as aerospace, automotive, textile and biomedical could be expected. The future work in FDM nanocomposites development is summarized as follows:

1. Continue to expand the nanocomposite portfolio. Most of the current matrix materials for composites used in FDM are based on several polymer types such as ABS and PLA. Undocumented or novel polymers need to be explored with good compatibility with FDM 3D printing. In addition, the building of processing-structure-property relationships for polymer nanocomposites in the context of FDM will facilitate the discovery of lighter, stronger and multifunctional materials, further expanding the capabilities of additive manufacturing.
2. Improve the FDM printing processes. Print parameters such as nozzle temperature, bed temperature, print speed, print orientation, layer height, etc., all play an important role in the performance of FDM-printed products. Currently, one of the main obstacles to the expansion of the FDM process is the slow printing speed [142], and how to improve the heat transfer properties of the printed material is an important factor for improving the FDM printing rate [143]. In addition, FDM is still not an alternative to traditional techniques (e.g., injection moulding) in terms of large-scale applications. The main issues including porosity, gaps between layers and the grating generated during the FDM process still widely exist.
3. Reduce the printing costs. For the manufacturing of small quantities of parts, FDM is often less costly than traditional manufacturing methods as it does not require the tooling process. However, the raw material for FDM printing is usually expensive. In addition, the printing filaments used for FDM require high dimensional accuracy to maintain good print quality. Due to the limited print resolution, post-processing is usually required for the final products. Compared to processes such as injection moulding, FDM generally consumes more energy and time.
4. Improve the recyclability of FDM printing materials. As more and more low-cost FDM devices enter the market, excessive waste of materials to produce low-value parts should be considered. From the perspective of materials development, reduced waste can be achieved by reducing support materials. Furthermore, one of the advantages of many thermoplastics is the recyclability. As FDM-manufactured parts are increasingly needed, their recyclability must be further explored to reduce the environmental burden of plastic pollution.

Additive manufacturing is a newly emerging technology compared to other traditional manufacturing methods and as such there are many technical issues that need to be investigated and broken through. For example, the lack of strength in the *z*-direction is due to the anisotropy of FDM printed parts. Although there are still many problems to be solved with FDM, we are confident that with the rapid development of the additive manufacturing field, FDM technology will be more widely used in the near future in aerospace, medical, automotive and other fields. In the meantime, more breakthroughs in the development of printable nanocomposites will emerge.

## Figures and Tables

**Table 1:** Summary of the most common thermoplastics used in the FDM process.

Polymer Type	Materials	Characterisation	References
ABS	Acrylonitrile butadiene styrene	Resistance to corrosive materials Low cost Withstand high temperature Easy to print	[53,54,55,56,57,58]
PLA	Polylactic Acid	Low cost Non-toxic Biodegradable Easy to print	[59,60,61,62,63,64,65]
PC	Polycarbonate	Transparent Temperature-resistant High resistance to impact	[66,67,68,69]
PEEK	Polyether-ether-ketone	Organic thermoplastic polymer Chemical-resistant Good lubricity	[70,71,72,73]
PETG	Polyethene terephthalate glycol	Chemical-resistant High processability	[74,75,76,77]
Nylon	Polyamide	Resistant to impact Heat-resistant High tensile strength	[78,79,80]
TPU	Thermoplastic polyurethane	Good lubricity Abrasion-resistant Low cost	[81,82]
PEI	Polyetherimide	Chemical-resistant Heat-resistant Dielectric	[83,84]
TPE	Thermoplastic elastomer	high recycling Easy to process Low cost	[85]
PP	Polypropylene	Good mechanical properties Good electrical insulation	[86,87]

## Data Availability

The data that support the findings of this study are available from the corresponding authors upon reasonable request.
